# Supplementary material for: Dietary protein source alters gut microbiota composition and function
Source: ISME J. 2025 Mar 21;19(1):wraf048. doi: 10.1093/ismejo/wraf048 (PMC12066410; doi:10.1093/ismejo/wraf048)
Supplement: SupplementaryResultsTablesandFigures_wraf048 [file supplementaryresultstablesandfigures_wraf048.pdf]

Supplementary Information

Dietary protein source alters gut microbiota composition and function

Authors: J. Alfredo Blakeley-Ruiz†*[1], Alexandria Bartlett†[1,2], Arthur S. McMillan[3], Ayesha Awan[1,3], Molly Vanhoy Walsh[1], Alissa K. Meyerhoffer[1], Simina Vintila[1], Jessie L. Maier[1], Tanner G. Richie[1], Casey M. Theriot[3], Manuel Kleiner*[1]

1. Department of Plant and Microbial Biology, College of Agricultural Sciences, North Carolina State University, Raleigh, NC, USA
2. Department of Molecular Genetics and Microbiology, Duke University, Durham, NC, USA
3. Department of Population Health and Pathobiology, College of Veterinary Medicine, North Carolina State University, Raleigh NC, USA

† These authors contributed equally to this manuscript

* Please refer correspondence to J. Alfredo Blakeley-Ruiz (jablakel@ncsu.edu) or Manuel Kleiner (manuel_kleiner@ncsu.edu)

Supplementary Results

Section A: Effect of dietary protein on gut microbiota composition

Hierarchical clustering of the proteinaceous biomass of species revealed distinct starting microbiota compositions for the group 1 and group 2 mice. This led us to surmise that the source of dietary protein altered the gut microbiota regardless of starting microbiota composition. To confirm this, we analyzed the group 1 and group 2 mice separately. We observed that they had similar alpha diversity and richness responses to dietary protein source regardless of mouse group and that the Bray-Curtis dissimilarity patterns were also similar (Fig. 1C and E; Supplementary Fig. S1A, B, and C). Principal component analysis showed the same separation as the hierarchical clustering between mouse group 1 and 2 along the first component (Supplementary Fig. S1D). Further analysis along the second and third components showed distinct dietary clusters between 1) soy, 2) T0, 3) yeast and egg white, and 4) casein, brown rice, and pea (Supplementary Fig. S1E). Principal component analysis on the separate mouse groups revealed the same cluster groups (Supplementary Fig. S1F and G), suggesting that the source of dietary protein alters the gut microbiota's composition regardless of starting microbiota composition.

We observed dynamic species abundance responses to different sources of dietary protein; these responses were in some cases consistent across mouse groups, whereas in other cases they were mouse group specific. For example, *Bacteroides thetaiotaomicron* increased in abundance across both mouse groups in the yeast and egg white diets (Fig. 1G; Supplementary Fig. S2), whereas the abundances of *Schaederella* sp. AB67-1 and *Lachnospiraceae bacterium* AB103-0 repeatedly increased in the presence of soy (Supplementary Fig. S2). Other changes in species abundance due to diet were mouse group specific. For example, *Lactobacillus johnsonii* AB1-1 increased in abundance in the pea and casein diets in the group 1 mice, whereas *Faecalibaculum rodentium* AB112-1 showed a similar pattern in the group 2 mice. *Oscillospiraceae* bacterium AB63-2 increased in abundance in the presence of the soy diets in the group 1 mice, whereas *Oscillospiraceae bacterium* AB54-6 followed a similar pattern in the group 2 mice. All of the abundant species were significantly different in abundance between at least two diets (Supplementary Fig. S2; linear mixed effect model ANOVA, $q < 0.05$).

Section B: Effect of dietary protein on microbiota function

The two most abundant broad functional categories of detected peptides were gene expression, which includes ribosomes, chaperones, and transcription related enzymes, and monosaccharide metabolism, which includes glycolysis and the metabolism of simple sugars other than glucose (Fig. 2). The microbial investment in gene expression enzymes increased in the yeast diet relative to all other diets (except standard chow) and decreased in the egg white diet relative to all other diets. This was driven by an overall increase in the abundance of ribosomal proteins in the yeast diet (Supplemental Fig. S3B). The abundance of ribosomal proteins has been suggested to be directly correlated with bacterial growth rates [1] suggesting

that overall bacterial growth rate is higher when mice were fed the yeast diet. This is further supported by the overall higher bacterial load in the yeast diet (Fig. 1D). In contrast, we observed gene expression proteins that assist with the synthesis and folding of proteins, e.g., elongation factors and chaperones, to be increased in the soy diets relative to some of the other diets (Supplementary Fig. S3B). We also observed an increase in stress proteins, including oxidative stress proteins, in the soy and casein diets relative to brown rice and yeast (Supplementary Fig. S3C). Oxidative stress interferes with the proper elongation and folding of diets, which could explain why chaperones and elongation factors are increased in the soy diet [2].

We observed a significant decrease in the abundance of monosaccharide metabolizing enzymes in the yeast and brown rice diets (Fig. 2). Most abundant in this category were the enzymes belonging to the energy pay-off phase of glycolysis (Supplementary Fig. S3D). However, many of the other functions within monosaccharide metabolism had different abundance patterns. For example, galactose and mannose metabolism enzymes were increased in the yeast diet (Supplementary Fig S3E), whereas along with galactose metabolism, we also observed a general increase in the abundance of fucose, glucosamine, and sialic acid metabolism in the egg white diet relative to other diets. Glucosamine and sialic acid metabolism were also increased in the casein and pea diets relative to other diets (Supplementary Fig S3E). Fucose, galactose, sialic acid, and acetylglucosamine are all components of mucin [3]. In summary, these results suggest that the source of dietary protein impacts sugar metabolism in the gut microbiota.

Two other broad functional categories that significantly changed in abundance due to dietary protein source were adhesion and motility proteins and fermentation proteins (Fig. 2). The microbiota invested significantly less in proteins categorized as adhesion and motility proteins in the yeast and egg diets. Flagellar proteins drove this result, which can be explained by the replacement of species from the class Clostridia with species from the class Bacteroidia because microbes in the phylum Bacteroidota usually do not have flagella [4]. The microbial investment in fermentation enzymes also decreased in the yeast and egg white diets (Fig. 2). We divided fermentation enzymes into three categories, ethanol producing, short-chain fatty acid (SCFA) producing, and lactic acid producing (Supplementary Fig. S3A). This categorization revealed that fermentation enzymes leading to SCFA metabolites were the primary drivers of the decrease in fermentation-related proteins in the yeast and egg white diets. Production of SCFAs has been previously linked to anti-inflammatory responses, which could suggest that the changes in microbiota composition observed in the yeast and egg white diets may be detrimental to host health [5,6].

Section C: Effect of brown rice and soy dietary protein on glycoside hydrolase abundance

Several glycoside hydrolases increased in the presence of the brown rice and soy diets. In the soy diet, the expression of glycoside hydrolases was reproducible, increasing in abundance each of the three times the mice were fed a soy diet (Fig. 4A) β-glucosidases and β-xylosidases from the CAZy protein family GH3 were increased in the soy diets, whereas the abundance of GH16s increased in the brown rice diet. The abundance change of GH16

classified enzymes in the brown rice diet was driven by a single protein from the uncultured species *Oscillospiraceae bacterium* AB63-2 that was only detected in the brown rice diet. In the presence of soy protein, but not brown rice protein, this bacterium reproducibly expressed different glycoside hydrolases including two β-glucosidases and one β-xylosidase from the protein family GH3 (Data Set 13). These results suggest that different protein sources generally affect the function and composition of the gut microbiota through the different glycan structures attached to their glycoproteins

Supplementary Table S1: Composition of nine fully defined diets. Diets were formulated in consultation with Envigo to be 20% or 40% protein by weight after evaluation of the protein source.

**Diet**	Teklad catalog number	Protein supplier	Protein (g/Kg)	Sucrose (g/Kg)	Corn starch (g/Kg)	Corn oil (g/Kg)	Cellulose (g/Kg)	Vitamin mix, Teklad (40060) (g/Kg)	Ethoxyquin, antioxidant (g/Kg)	Mineral mix, Ca-P deficient (79055) (g/Kg)	Calcium phosphate dibasic (g/Kg)	Calcium carbonate (g/Kg)
**20% soy**	TD.190249	Teklad	230.00	436.10	200.00	52.30	37.86	10.00	0.01	13.37	15.26	5.10
**20% casein**	TD.190254	Teklad	230.00	434.70	200.00	52.30	37.86	10.00	0.01	13.37	16.66	5.10
**20% brown rice**	TD.190252	Swanson	260.00	400.43	200.00	54.60	37.86	10.00	0.01	13.37	23.72	0.00
**40% soy**	TD.190250	Teklad	460.00	211.79	200.00	50.00	37.86	10.00	0.01	13.37	6.80	10.18
**20% yeast**	TD.190253	Teklad	400.00	279.06	200.00	42.60	37.86	10.00	0.01	13.27	0.00	17.10
**40% casein**	TD.190255	Teklad	460.00	208.96	200.00	50.00	37.86	10.00	0.01	13.37	9.60	10.20
**20% pea**	TD.190251	NAKED	222.00	438.00	200.00	50.82	37.86	10.00	0.01	13.37	19.72	8.00
**20% egg white solids, spray-dried**	TD.190256	Teklad	248.00	412.67	200.00	54.60	37.86	10.00	0.01	13.37	22.54	0.50
**20% chicken bone broth**	TD.190257	Ancient Nutrition	220.00	450.33	200.00	44.70	37.86	10.00	0.01	13.37	23.73	0.00

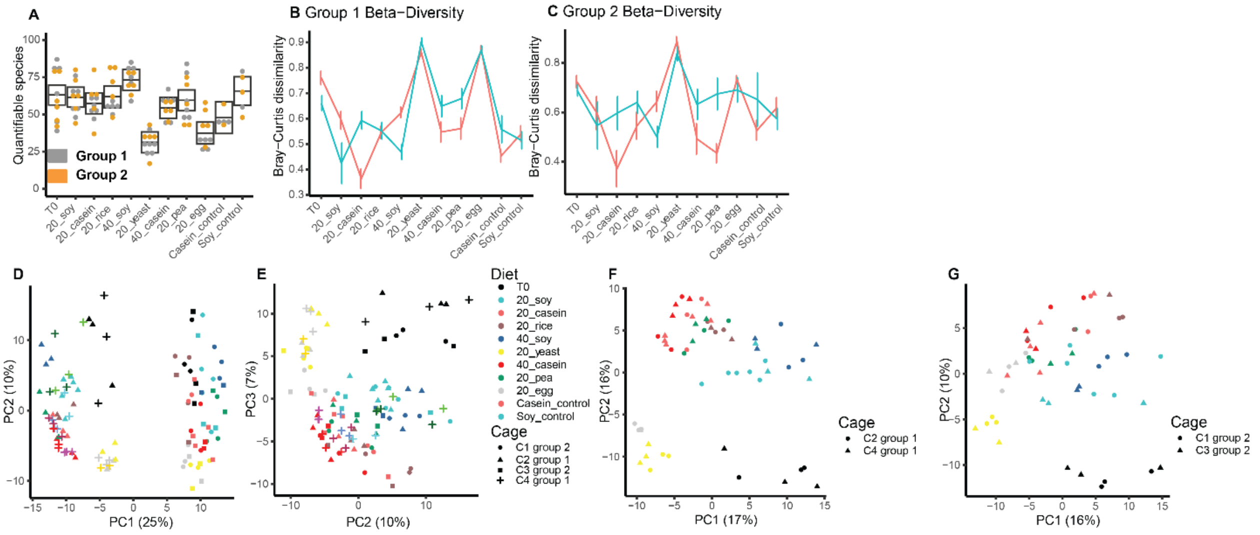

Supplementary Figure S1: Source of dietary protein alters the gut microbiota's composition. (A) depicts the average number of quantifiable species per diet; boxes represent the 95% confidence interval based on linear mixed effects models (Data Set 2). (B) and (C) depict the Bray-Curtis dissimilarity between 20% soy diets (teal) or 20% casein diets (red) and all other diets for group 1 and group 2, respectively. (D) and (E) first, second, and third principal components of microbiota composition based on species level metaproteomic proteinaceous biomass. (F) and (G) first and second components of microbiota composition based on species level metaproteomics proteinaceous biomass for the group 1 and group 2 mice, respectively.

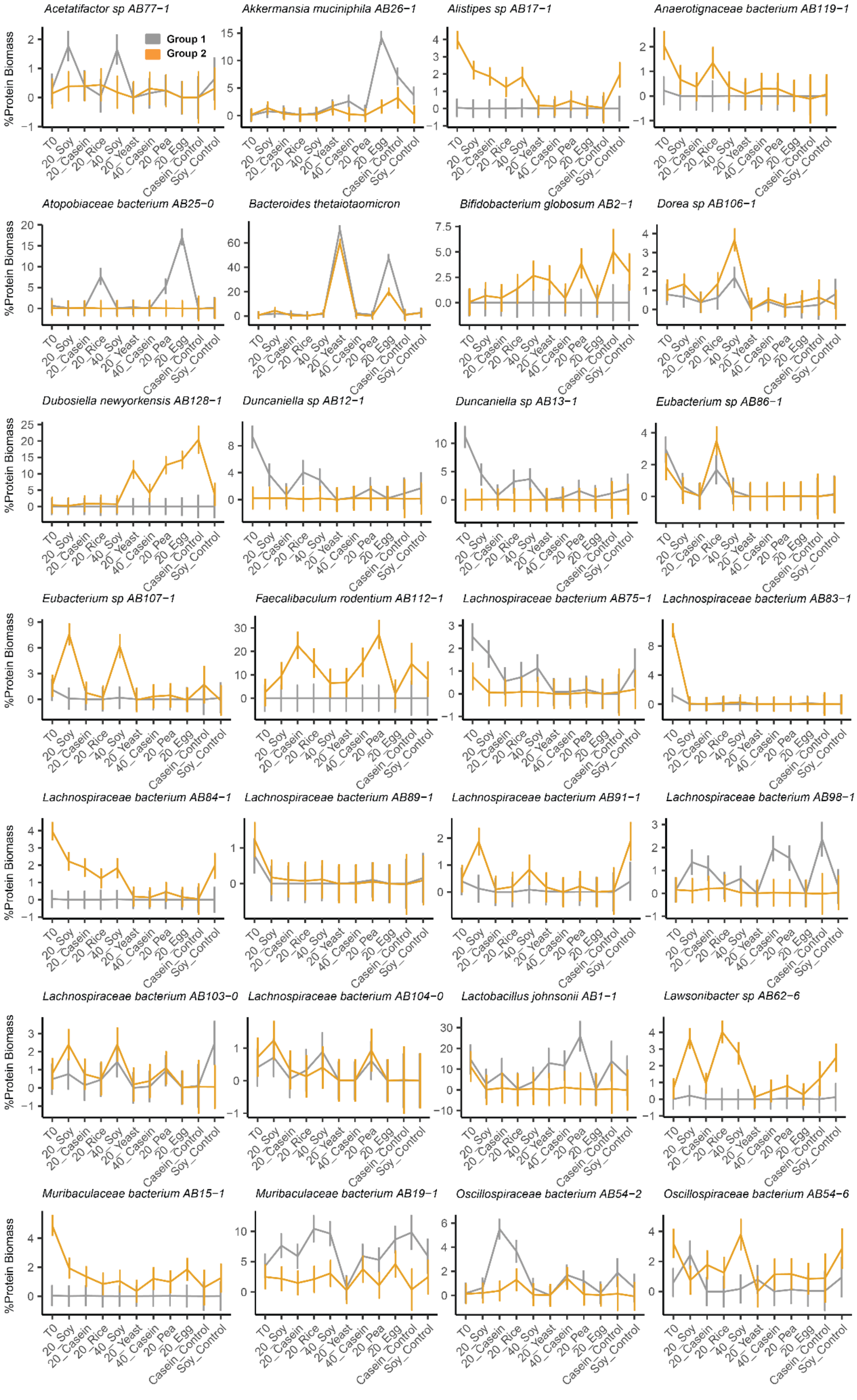
Acetatifactor sp AB77−1
Akkermansia muciniphila AB26−1
Alistipes sp AB17−1
Anaerotignaceae bacterium AB119−1
Atopobiaceae bacterium AB25−0
Bacteroides thetaiotaomicron
Bifidobacterium globosum AB2−1
Dorea sp AB106−1
Dubosiella newyorkensis AB128−1
Duncaniella sp AB12−1
Duncaniella sp AB13−1
Eubacterium sp AB86−1
Eubacterium sp AB107−1
Faecalibaculum rodentium AB112−1
Lachnospiraceae bacterium AB75−1
Lachnospiraceae bacterium AB83−1
Lachnospiraceae bacterium AB84−1
Lachnospiraceae bacterium AB89−1
Lachnospiraceae bacterium AB91−1
Lachnospiraceae bacterium AB98−1
Lachnospiraceae bacterium AB103−0
Lachnospiraceae bacterium AB104−0
Lactobacillus johnsonii AB1−1
Lawsonibacter sp AB62−6
Muribaculaceae bacterium AB15−1
Muribaculaceae bacterium AB19−1
Oscillospiraceae bacterium AB54−2
Oscillospiraceae bacterium AB54−6
Group 1
Group 2
%Protein Biomass
T0
20_Soy
20_Casein
20_Rice
40_Soy
20_Yeast
40_Casein
20_Pea
20_Egg
Casein_Control
Soy_Control

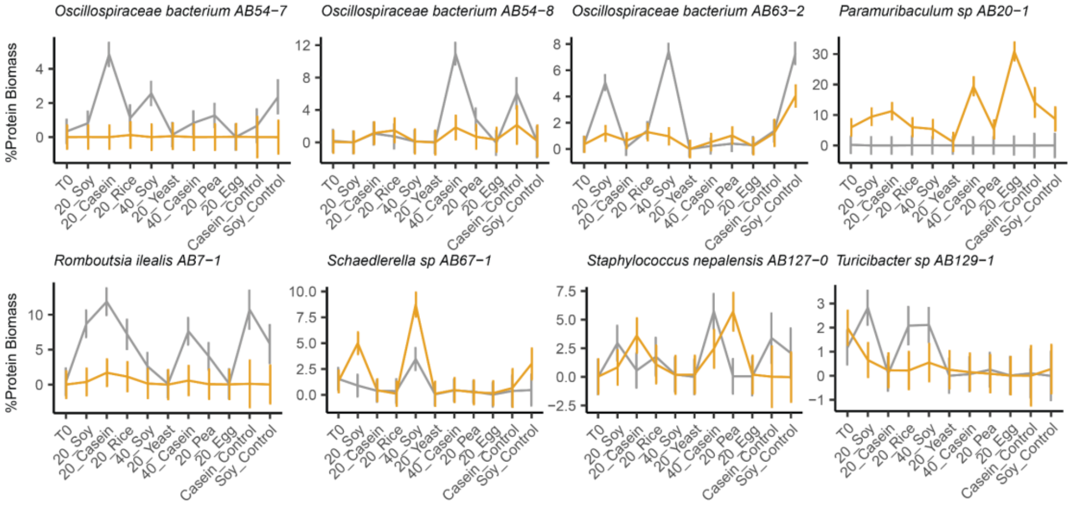

Supplementary Figure S2: Abundances of the 36 most abundant species across diets and mouse groups. Line plots depicting the average abundance of bacterial species in the group 1 mice (gray) and group 2 mice (orange) across all diets. Abundances were determined from metaproteomic data using a biomass assessment method [7]. Error bars represent the 95% confidence interval of the mean using mixed effects modeling. Species were defined as abundant if they represented at least 5% of the microbial protein mass in one sample.

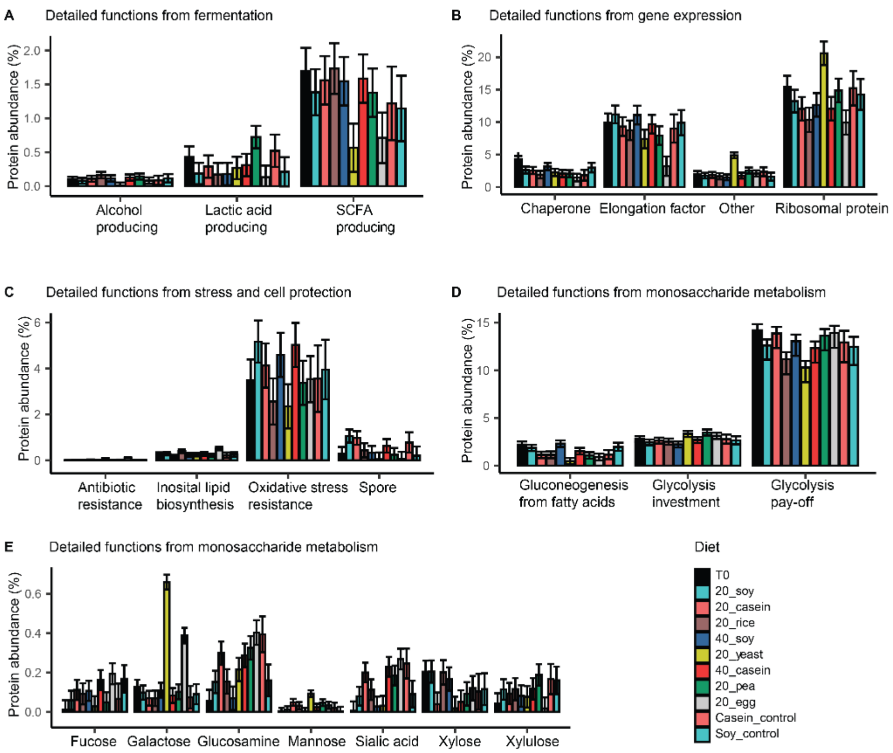

Supplementary Figure S3: Abundance of detailed functional categories associated with fermentation, gene expression, stress and cell protection, and monosaccharide metabolism. The mean protein abundance (% of total microbial proteins) per sample of each detailed function based on a complete linear mixed effects model. Error bars represent 95% confidence intervals and error bars that do not overlap indicate significant abundance differences. (A) Detailed functions that make up the fermentation broad functional category. (B) Detailed functions that make up the gene expression functional category. (C) Detailed functions that make up the stress and cell protection functional category. (D) The most abundant detailed functions that make up the monosaccharide metabolism functional category. (E) Select detailed functions that make up part of the monosaccharide metabolism category (Data Sets 6, 7 and 8).

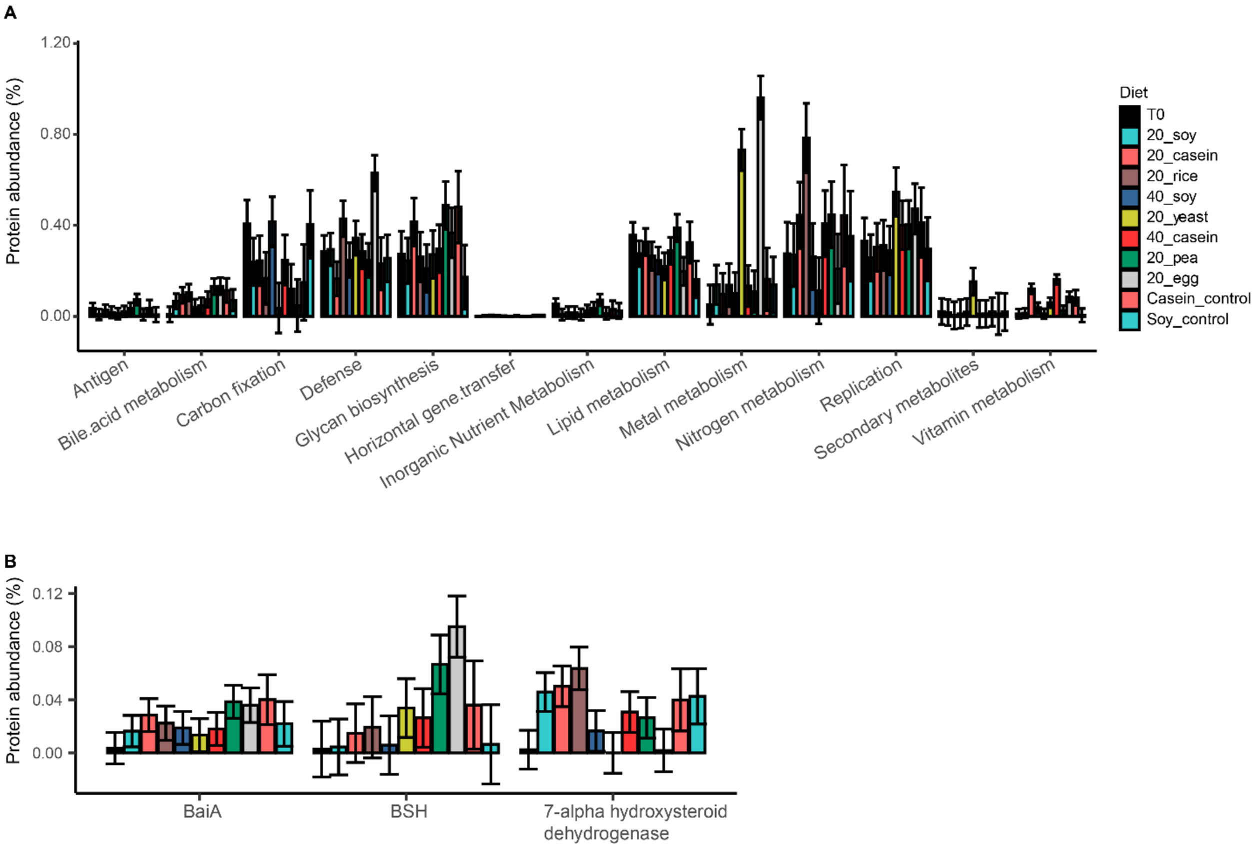

Supplementary Figure S4: Effect of dietary protein source on lower abundance protein functions. The mean protein abundance (% of total microbial proteins) per sample of each detailed function based on a complete linear mixed effects model. Error bars represent 95% confidence intervals and error bars that do not overlap indicate significant abundance differences. (A) Broad functional categories that represented less than 1% of the microbial protein abundance. (B) Consensus names of specific bile acid modifying enzymes (Data Sets 6, 7 and 8).

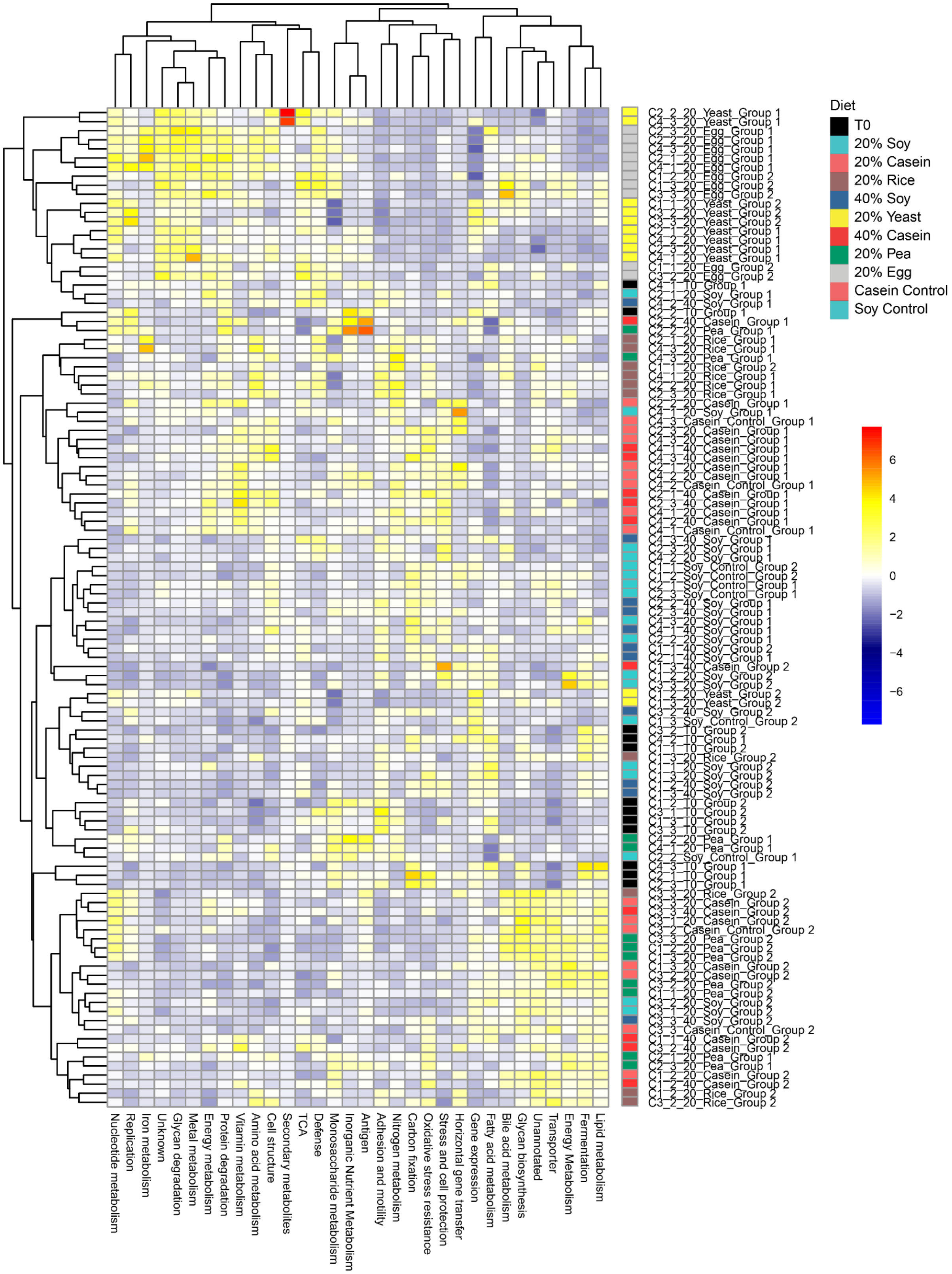

Supplementary Figure S5: Hierarchical clustering of broad functional categories. Tabulated abundances of broad functional categories (Data Set 6) were *Z* scored by feature and then clustered using the ward.D2 method using the pheatmap package in R.

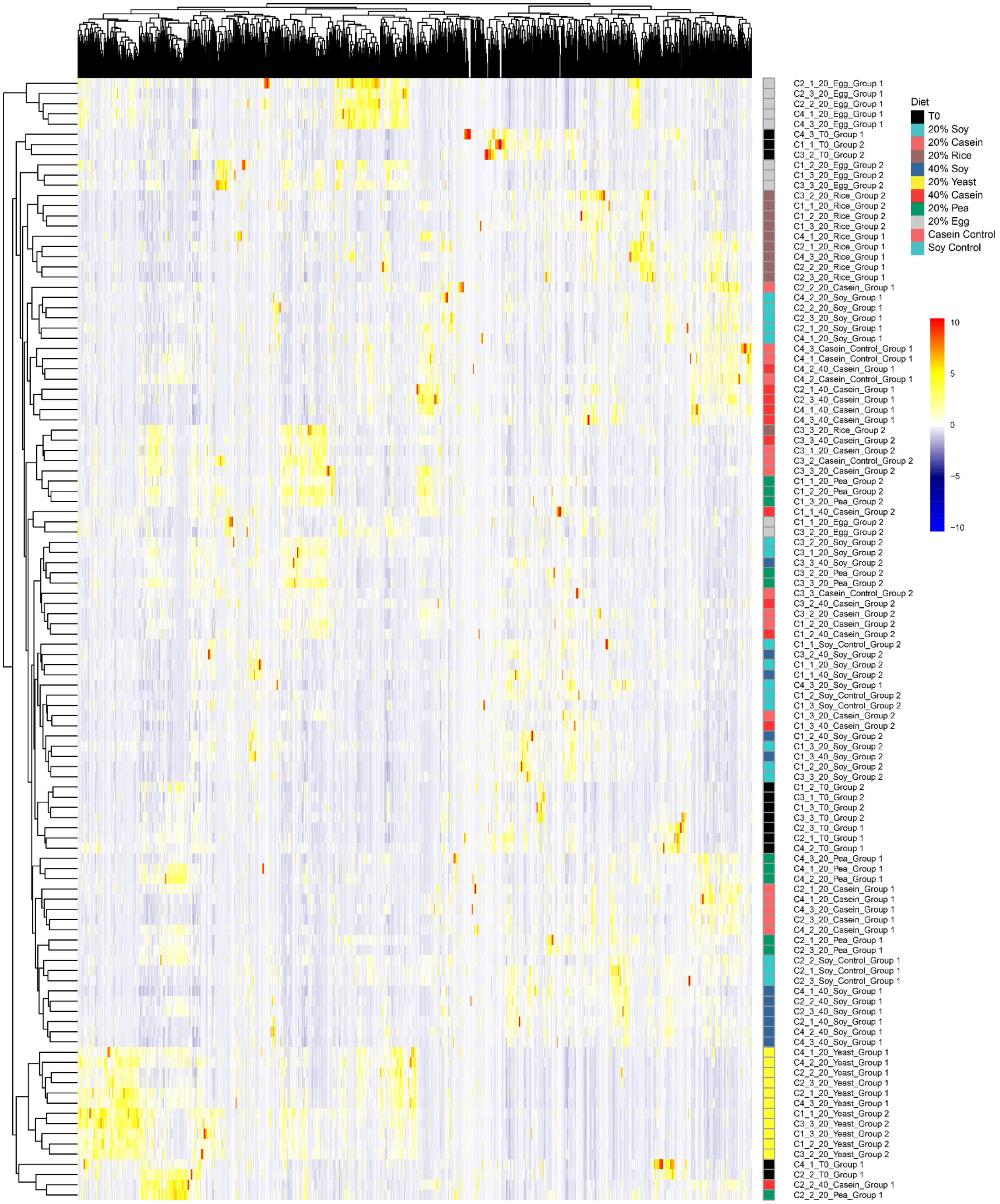

Supplementary Figure S6: Hierarchical clustering of consensus enzyme names. Tabulated abundances of consensus enzyme names (Data Set 6) were *Z* scored by feature and then clustered using the ward.D2 method using the pheatmap package in R.

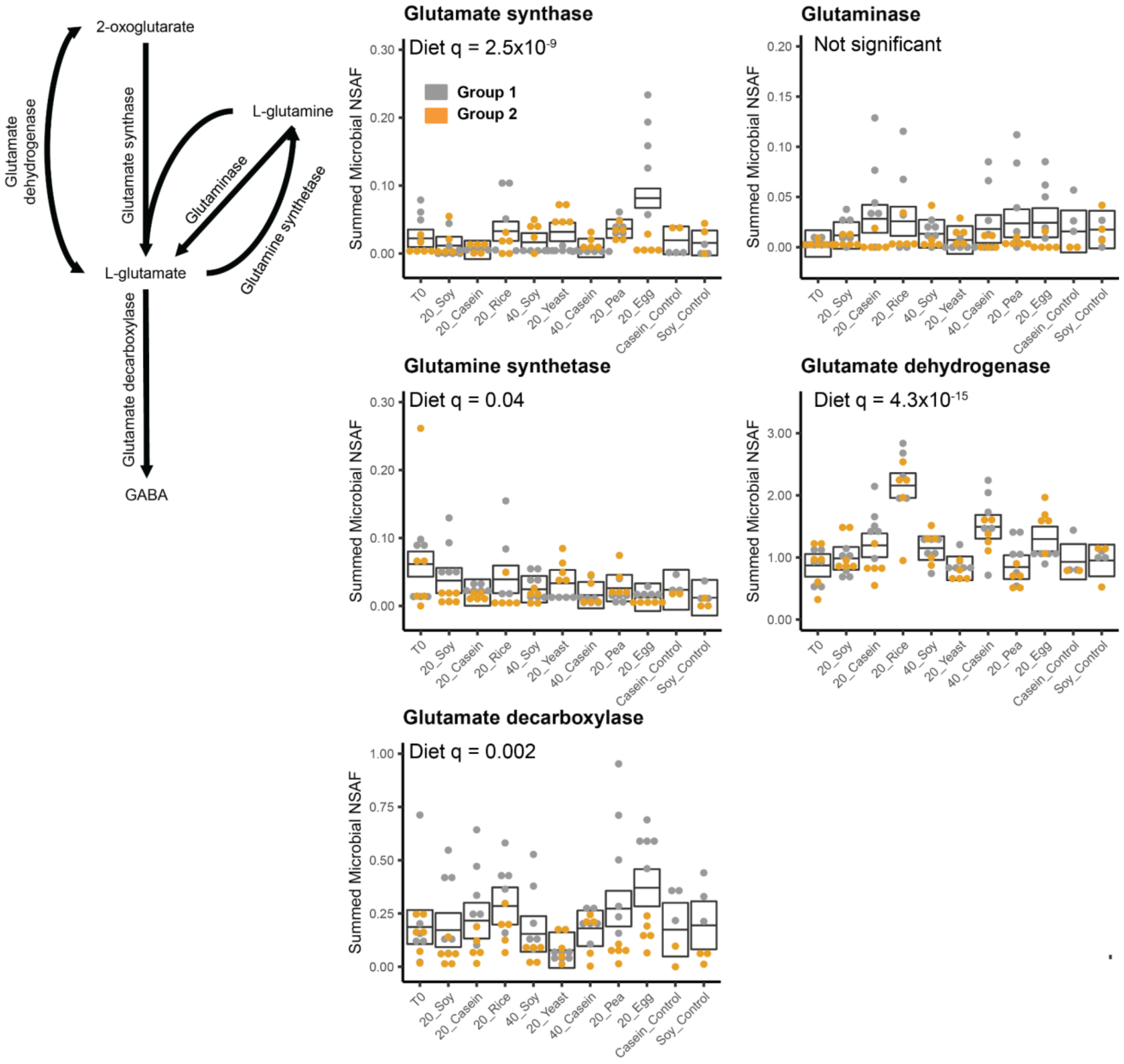

Supplementary Figure S7: Changes in glutamate and glutamine metabolism due to source of dietary protein. A reconstruction of the pathways involved in glutamate and glutamine metabolism based on enzymes detected in our metaproteomes. Box plots represent the aggregate abundance of the specific enzymes involved in the pathway. The boxes represent the 95% confidence interval of the mean (line) for each diet from a complete mixed effects model, and the *q* values represent the FDR controlled *P* values for the diet factor from an ANOVA on these models ($q<0.05$ indicates significance) (Data Sets 11-12).

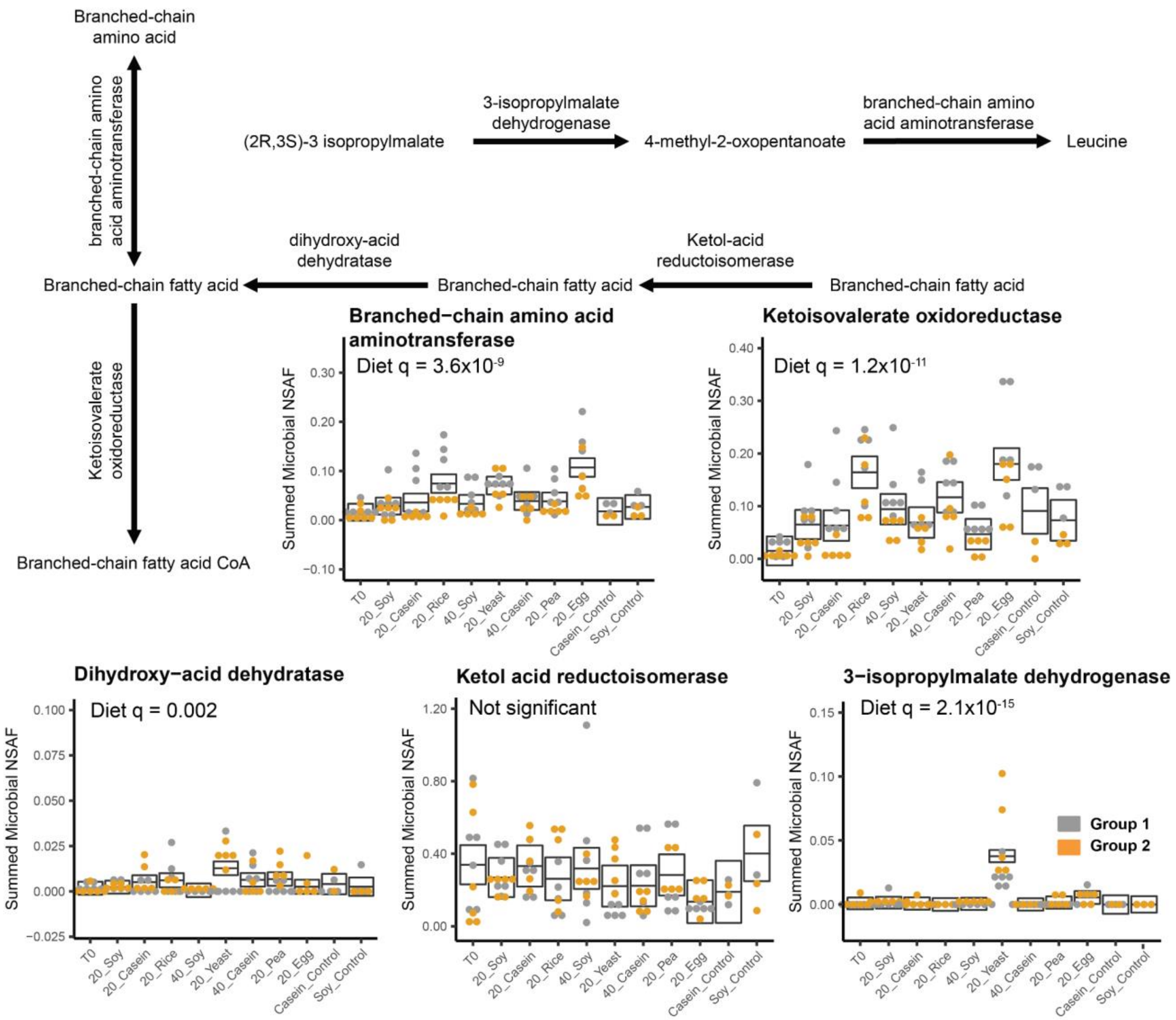

Supplementary Figure S8: Changes in branched-chain amino acid metabolism due to source of dietary protein. A reconstruction of the pathways involved in valine, leucine, and isoleucine metabolism based on enzymes detected in our metaproteomes. With the exception of leucine synthesis enzymes, the same enzymes act on all three of these amino acids so we did not try to distinguish them. Box plots represent the aggregate abundance of the specific enzymes involved in the pathway. The boxes represent the 95% confidence interval of the mean (line) for each diet from a complete mixed effects model, and the *q* values represent the FDR controlled *P* values for the diet factor from an ANOVA on these models ($q<0.05$ indicates significance) (Data Sets 11-12).

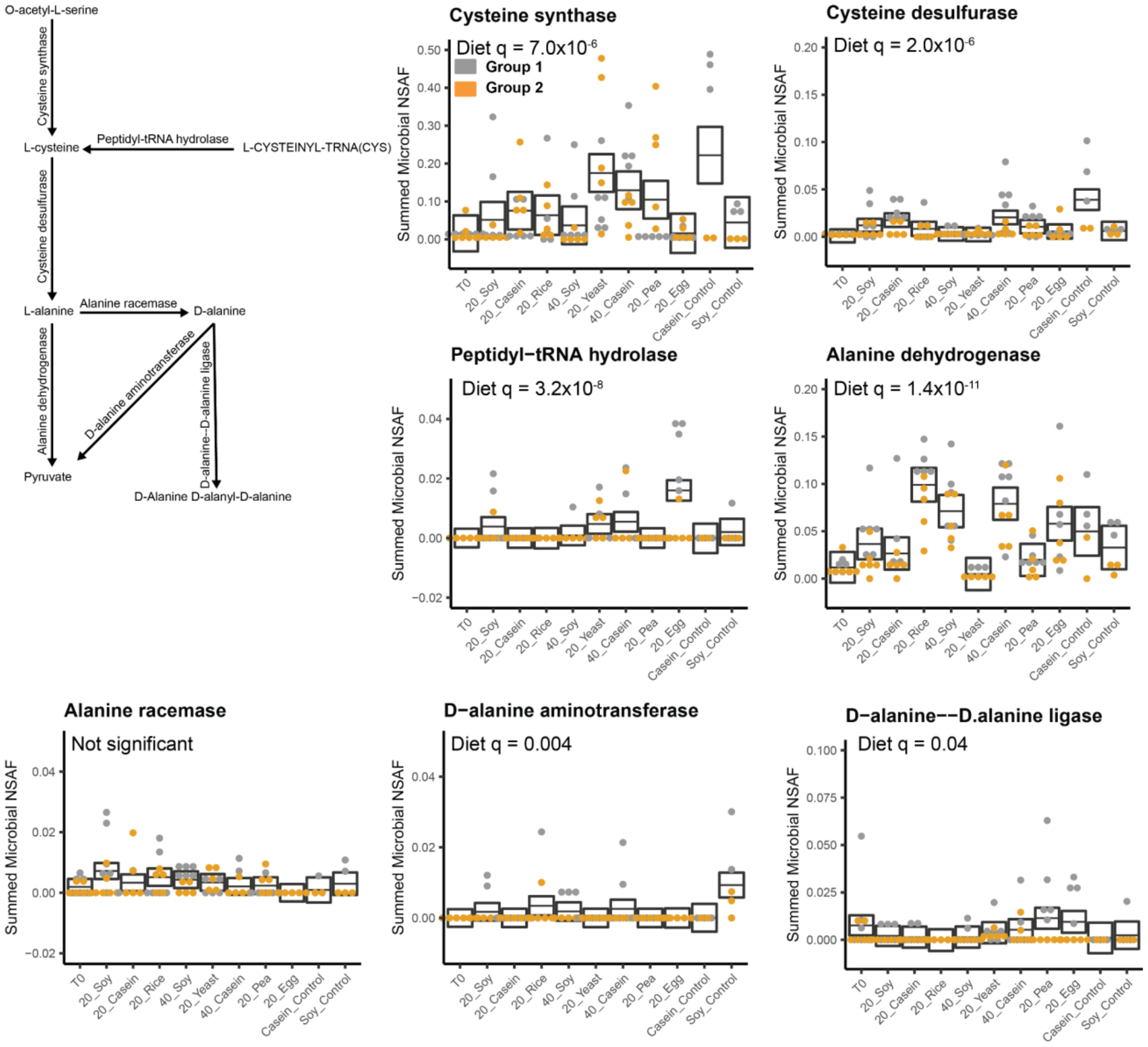

Supplementary Figure S9: Changes in cysteine and alanine metabolism due to source of dietary protein. A reconstruction of the pathways involved in cysteine and alanine metabolism based on enzymes detected in our metaproteomes. Box plots represent the aggregate abundance of the specific enzymes involved in the pathway. The boxes represent the 95% confidence interval of the mean (line) for each diet from a complete mixed effects model, and the *q* values represent the FDR controlled *P* values for the diet factor from an ANOVA on these models ($q<0.05$ indicates significance) (Data Sets 11-12).

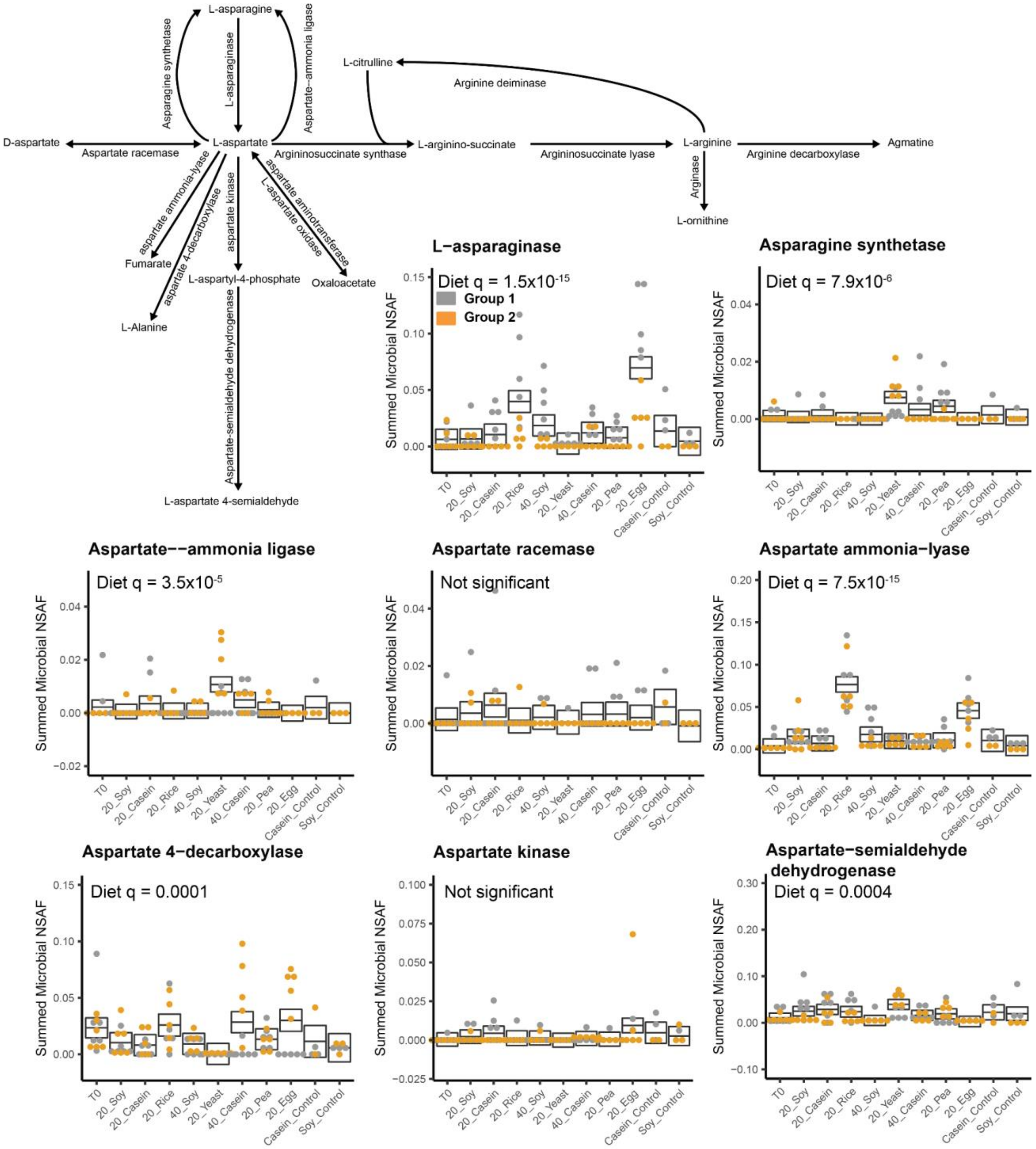

Supplementary Figure S10: Changes in asparagine, aspartate, and arginine metabolism due to source of dietary protein part 1. A reconstruction of the pathways involved in asparagine, aspartate, and arginine metabolism based on enzymes detected in our metaproteomes. Box plots represent the aggregate abundance of the specific enzymes involved in the pathway. The boxes represent the 95% confidence interval of the mean (line) for each diet from a complete mixed effects model, and the *q* values represent the FDR controlled *P* values for the diet factor from an ANOVA on these models ($q<0.05$ indicates significance) (Data Sets 11-12).

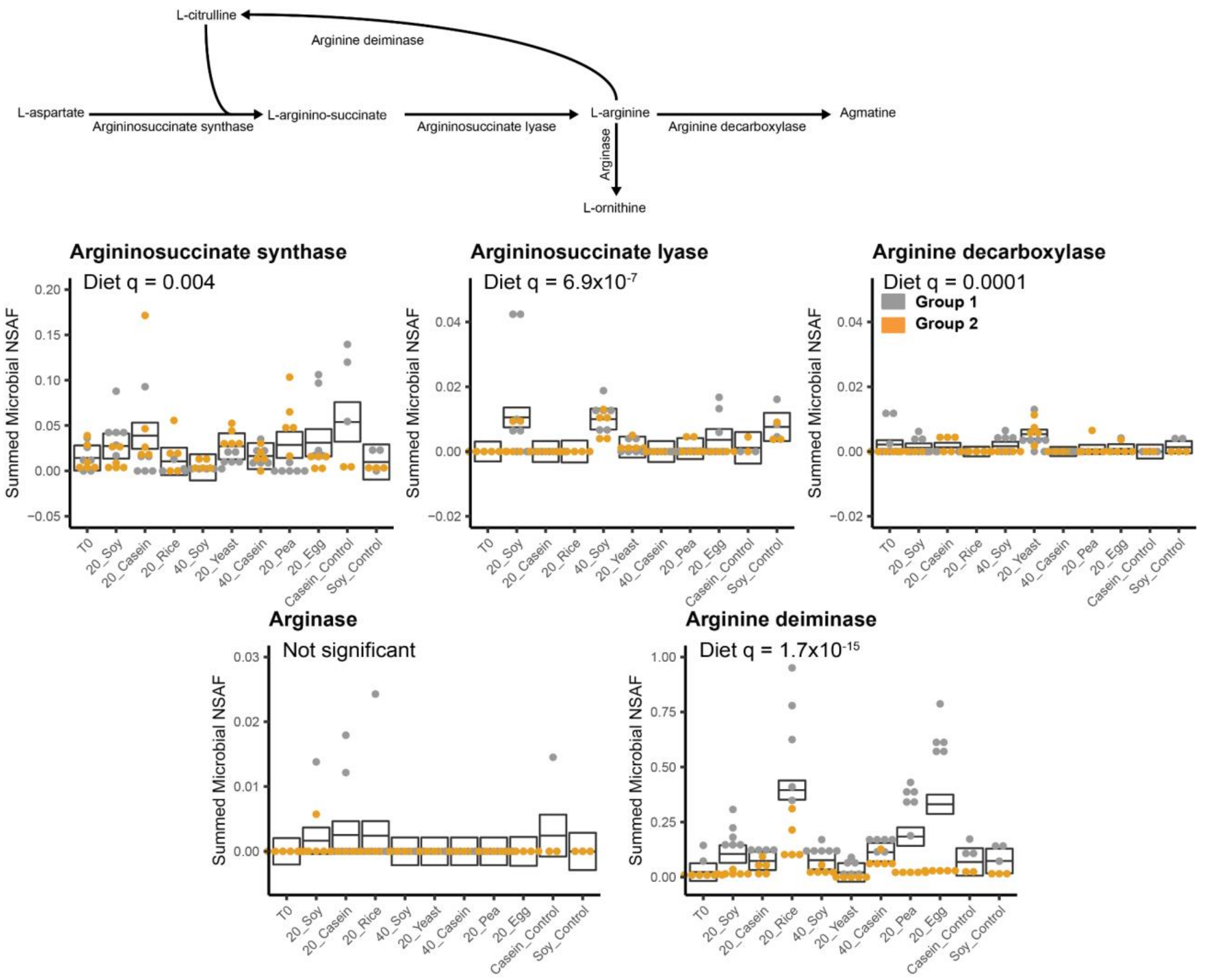

Supplementary Figure S11: Changes in asparagine, aspartate, and arginine metabolism due to source of dietary protein part 2. A reconstruction of the pathways involved in asparagine, aspartate, and arginine metabolism based on enzymes detected in our metaproteomes. Box plots represent the aggregate abundance of the specific enzymes involved in the pathway. The boxes represent the 95% confidence interval of the mean (line) for each diet from a complete mixed effects model, and the *q* values represent the FDR controlled *P* values for the diet factor from an ANOVA on these models ($q<0.05$ indicates significance) (Data Sets 11-12).

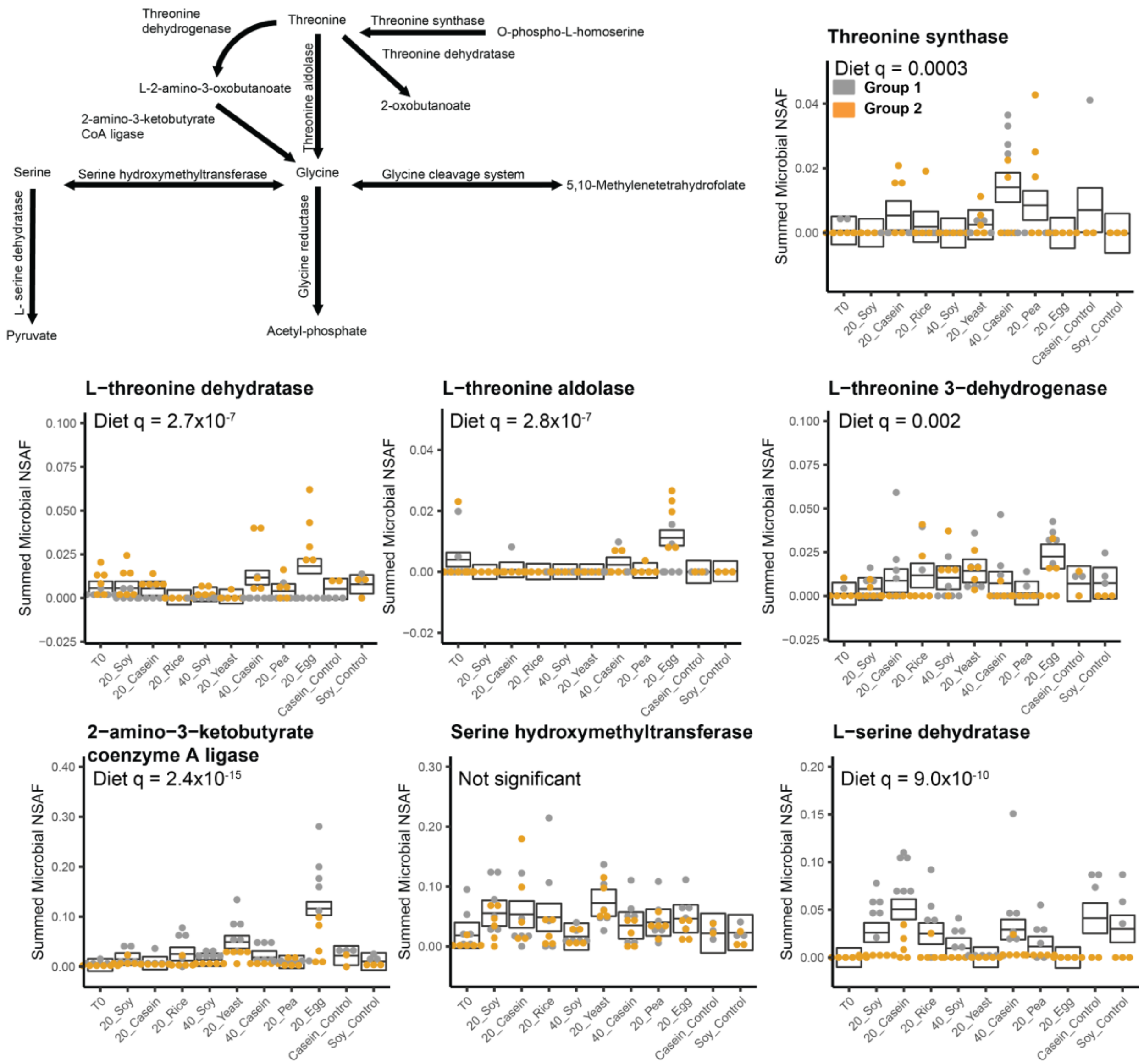

Supplementary Figure S12: Changes in threonine, glycine, and serine metabolism due to source of dietary protein part 1. A reconstruction of the pathways involved in threonine, glycine, and serine metabolism based on enzymes detected in our metaproteomes. Box plots represent the aggregate abundance of the specific enzymes involved in the pathway. The boxes represent the 95% confidence interval of the mean (line) for each diet from a complete mixed effects model, and the *q* values represent the FDR controlled *P* values for the diet factor from an ANOVA on these models ($q<0.05$ indicates significance) (Data Sets 11-12).

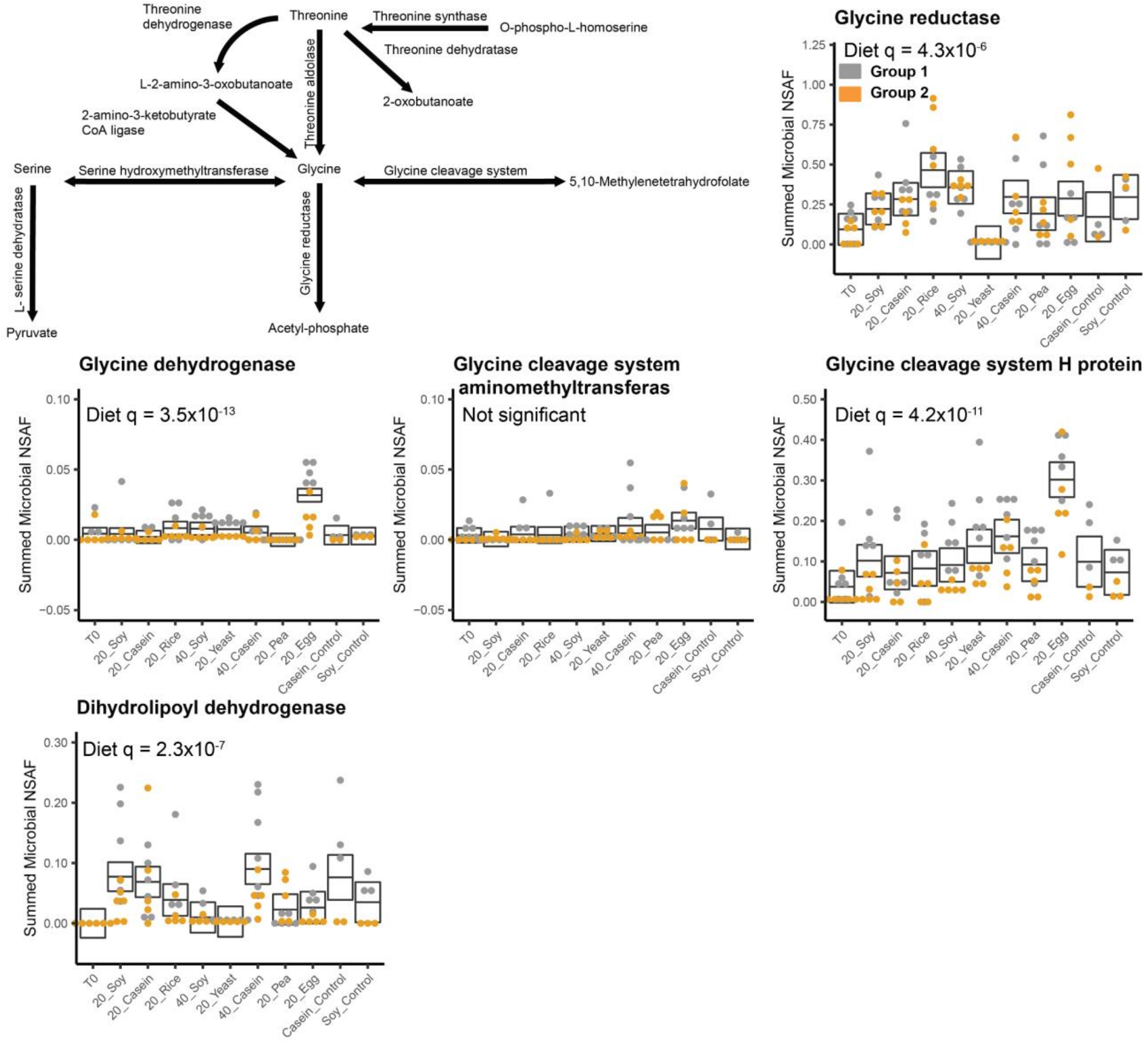

Supplementary Figure S13: Changes in threonine, glycine, and serine metabolism due to source of dietary protein part 2. A reconstruction of the pathways involved in threonine, glycine, and serine metabolism based on enzymes detected in our metaproteomes. Box plots represent the aggregate abundance of the specific enzymes involved in the pathway. The boxes represent the 95% confidence interval of the mean (line) for each diet from a complete mixed effects model, and the *q* values represent the FDR controlled *P* values for the diet factor from an ANOVA on these models (q<0.05 indicates significance) (Data Sets 11-12).

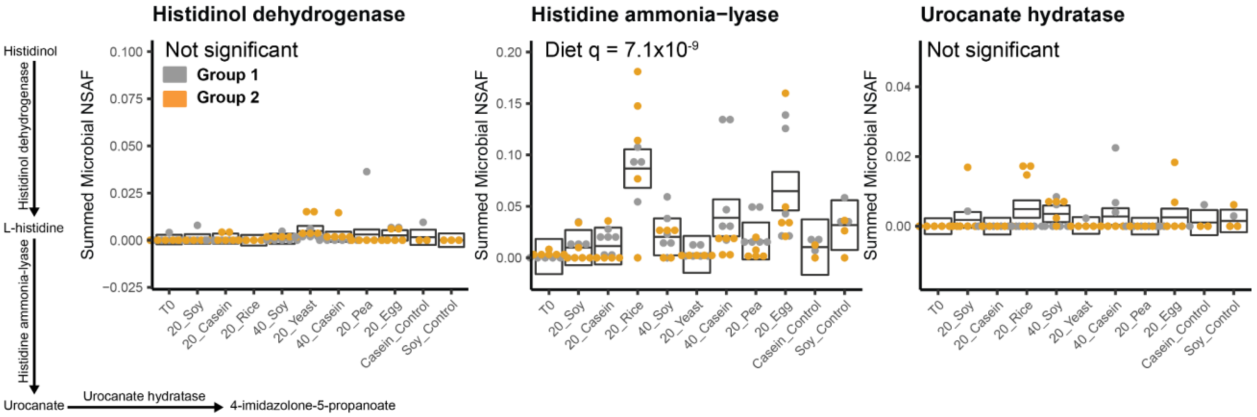

Supplementary Figure S14: Changes in histidine metabolism due to source of dietary protein. A reconstruction of the pathways involved in histidine metabolism based on enzymes detected in our metaproteomes. Box plots represent the aggregate abundance of the specific enzymes involved in the pathway. The boxes represent the 95% confidence interval of the mean (line) for each diet from a complete mixed effects model, and the *q* values represent the FDR controlled *P* values for the diet factor from an ANOVA on these models (q<0.05 indicates significance) (Data Sets 11-12).

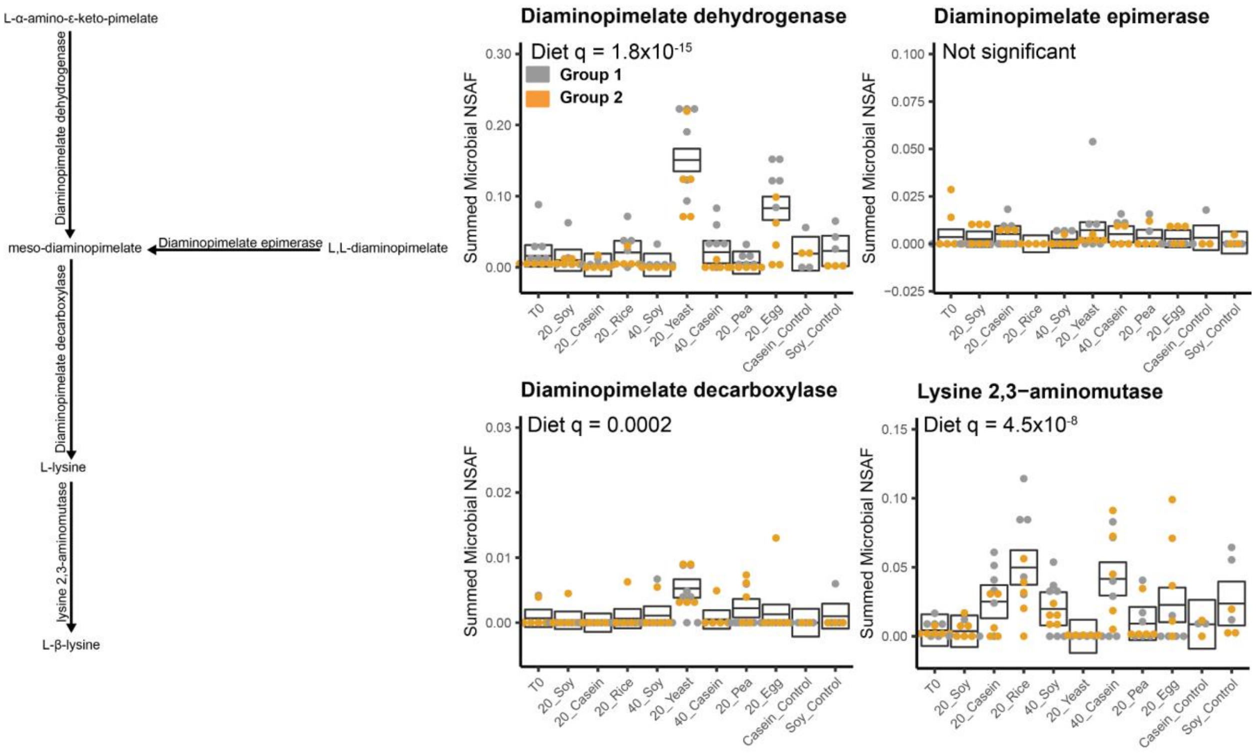

Supplementary Figure S15: Changes in lysine metabolism due to source of dietary protein. A reconstruction of the pathways involved in lysine metabolism based on enzymes detected in our metaproteomes. Box plots represent the aggregate abundance of the specific enzymes involved in the pathway. The boxes represent the 95% confidence interval of the mean (line) for each diet from a complete mixed effects model, and the *q* values represent the FDR controlled *P* values for the diet factor from an ANOVA on these models (*q*<0.05 indicates significance) (Data Sets 11-12).

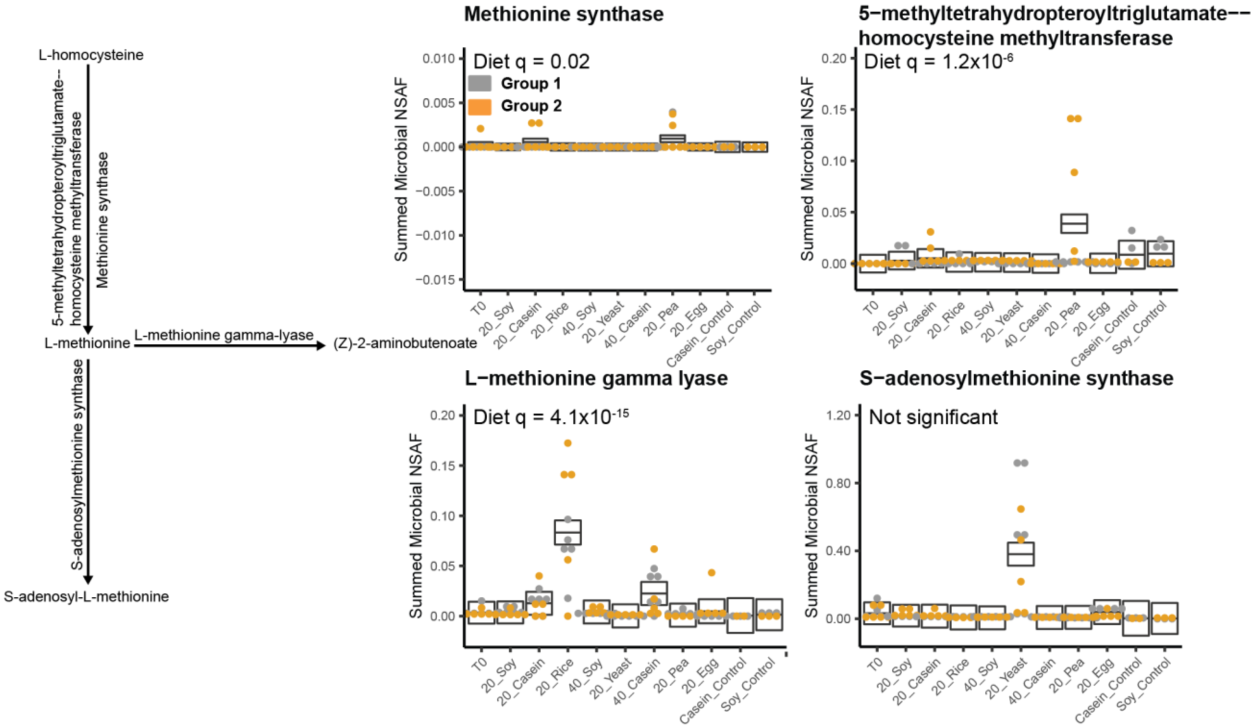

Supplementary Figure S16: Changes in methionine metabolism due to source of dietary protein. A reconstruction of the pathways involved in methionine metabolism based on enzymes detected in our metaproteomes. Box plots represent the aggregate abundance of the specific enzymes involved in the pathway. The boxes represent the 95% confidence interval of the mean (line) for each diet from a complete mixed effects model, and the *q* values represent the FDR controlled *P* values for the diet factor from an ANOVA on these models (q<0.05 indicates significance) (Data Sets 11-12).

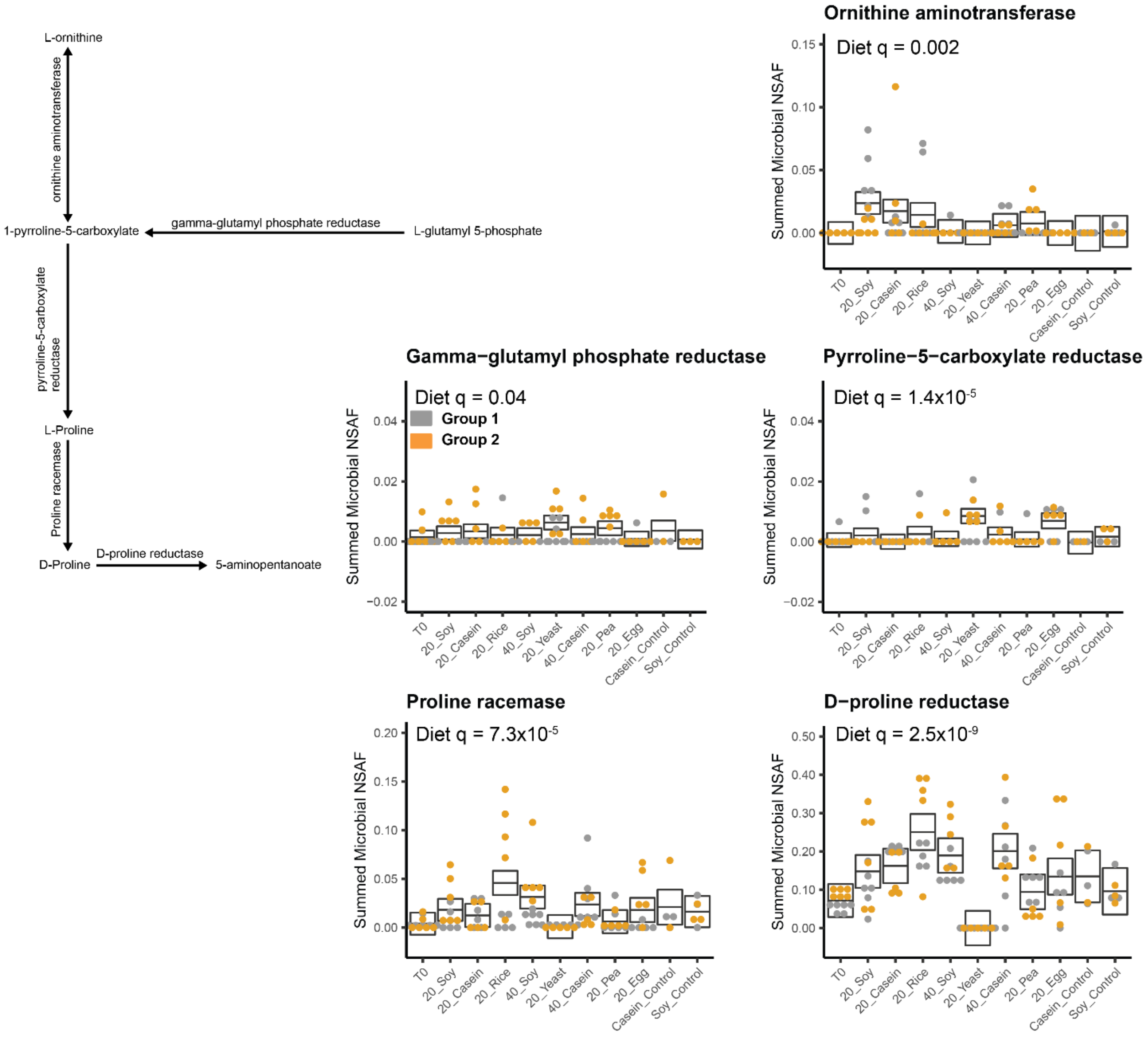

Supplementary Figure S17: Changes in proline metabolism due to sources of dietary protein. A reconstruction of the pathways involved in proline metabolism based on enzymes detected in our metaproteomes. Box plots represent the aggregate abundance of the specific enzymes involved in the pathway. The boxes represent the 95% confidence interval of the mean (line) for each diet from a complete mixed effects model, and the *q* values represent the FDR controlled *P* values for the diet factor from an ANOVA on these models ($q<0.05$ indicates significance) (Data Sets 11-12).

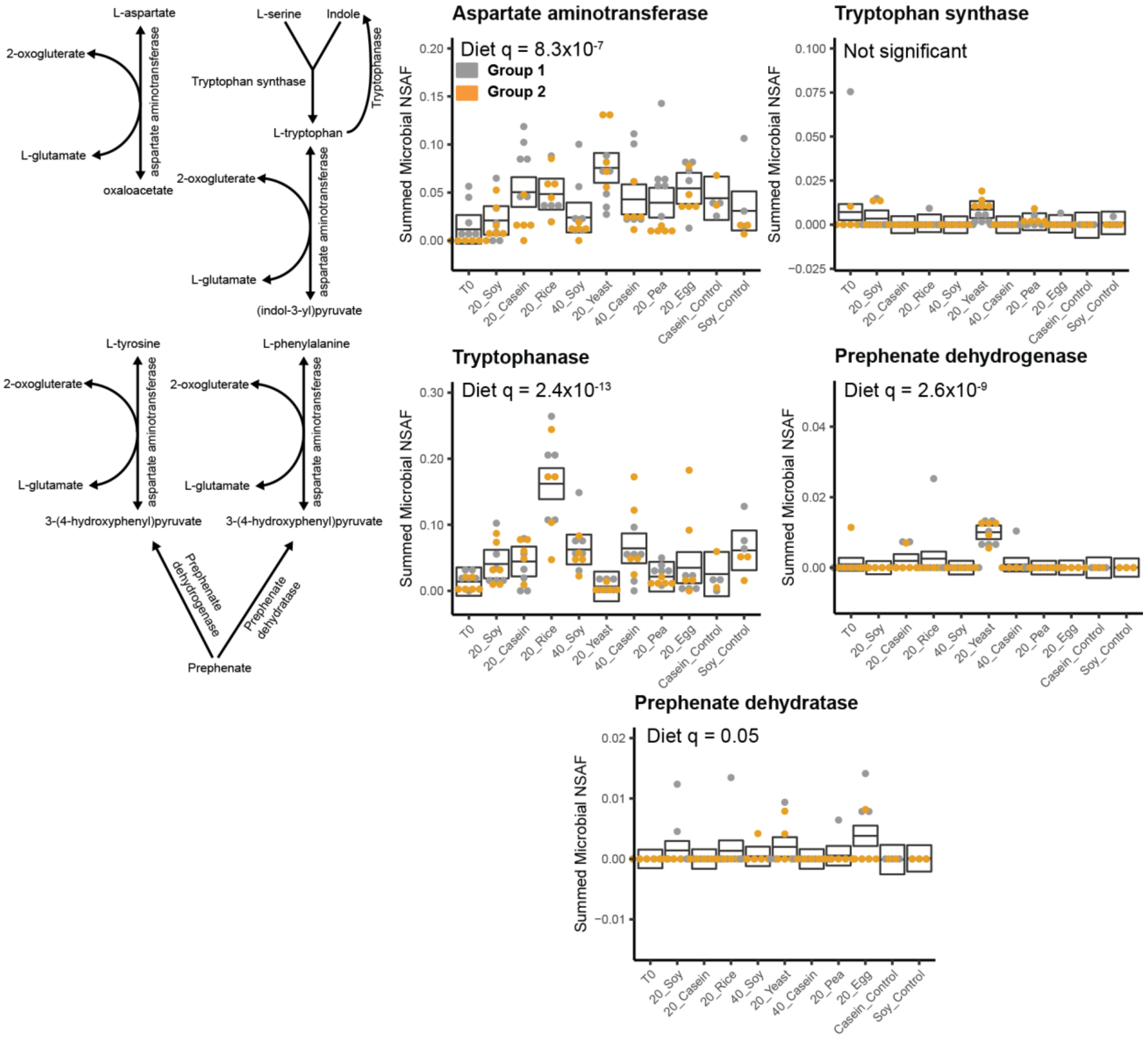

Supplementary Figure S18: Changes in aspartate, serine, tryptophan, tyrosine, and phenylalanine metabolism due to source of dietary protein. A reconstruction of the pathways involved in aspartate, serine, tryptophan, tyrosine, and phenylalanine metabolism based on enzymes detected in our metaproteomes. Box plots represent the aggregate abundance of the specific enzymes involved in the pathway. The boxes represent the 95% confidence interval of the mean (line) for each diet from a complete mixed effects model, and the *q* values represent the FDR controlled *P* values for the diet factor from an ANOVA on these models ($q<0.05$ indicates significance) (Data Sets 11-12).

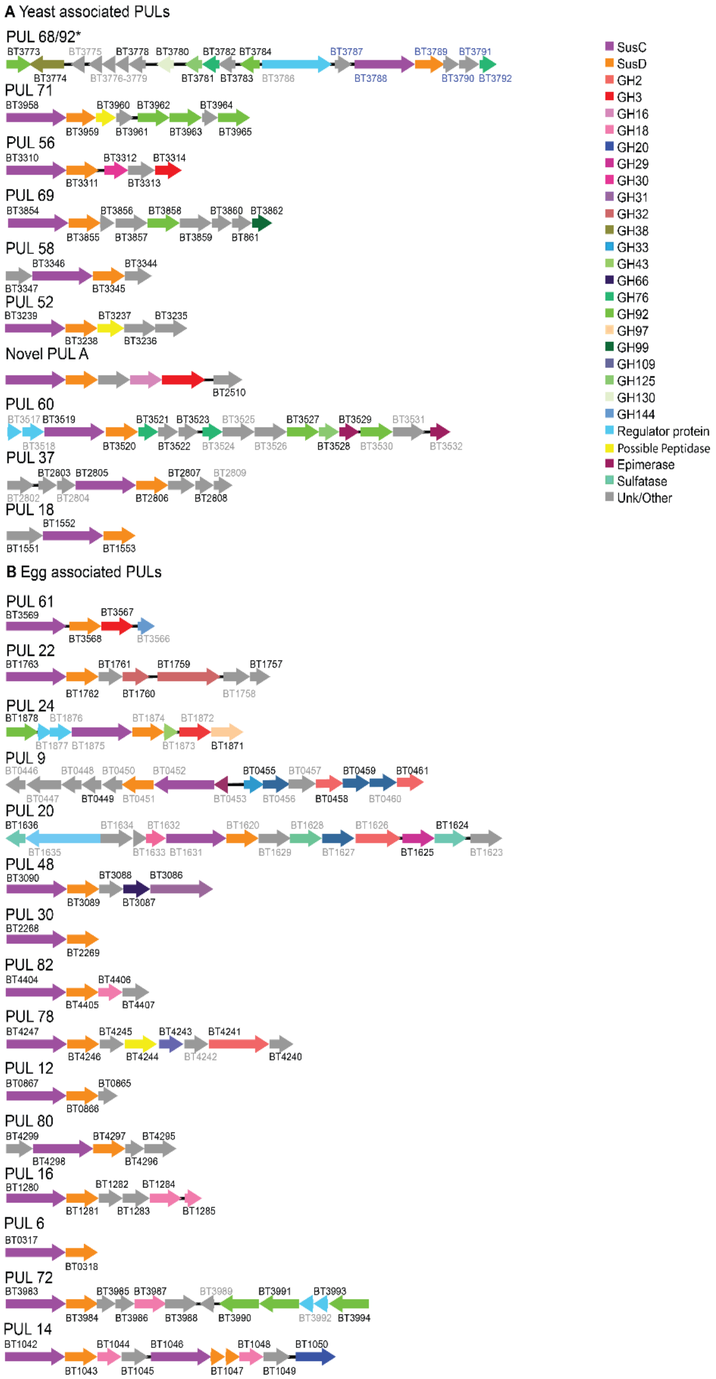

Supplementary Figure S19: Distinct polysaccharide utilization loci (PULs) are expressed by B. thetaiotaomicron between the egg white and yeast diet. Graphical representation of PUL gene neighborhoods detected in the metaproteome. PUL identifiers are the literature derived PUL identifiers from PULDB [8], but the PUL structure was verified in RAST using the B. thetaiotaomicron genome assembled from our metagenome. Metagenome identifiers were cross referenced to BT numbers from previous PUL papers. If the BT number is black, it is detected in the metaproteome, if gray it is not detected in our metaproteome but detected in our genome, and if blue it means that we did not have those genes in our genome but instead detected homologs with the exact same gene neighborhood structure and similar gene percent identity. (A) PUL operons detected in our metaproteome that were increased in the yeast diet relative to the egg white diet. (B) PUL operons detected in our metaproteome that were increased in the egg white diet relative to the yeast diet.

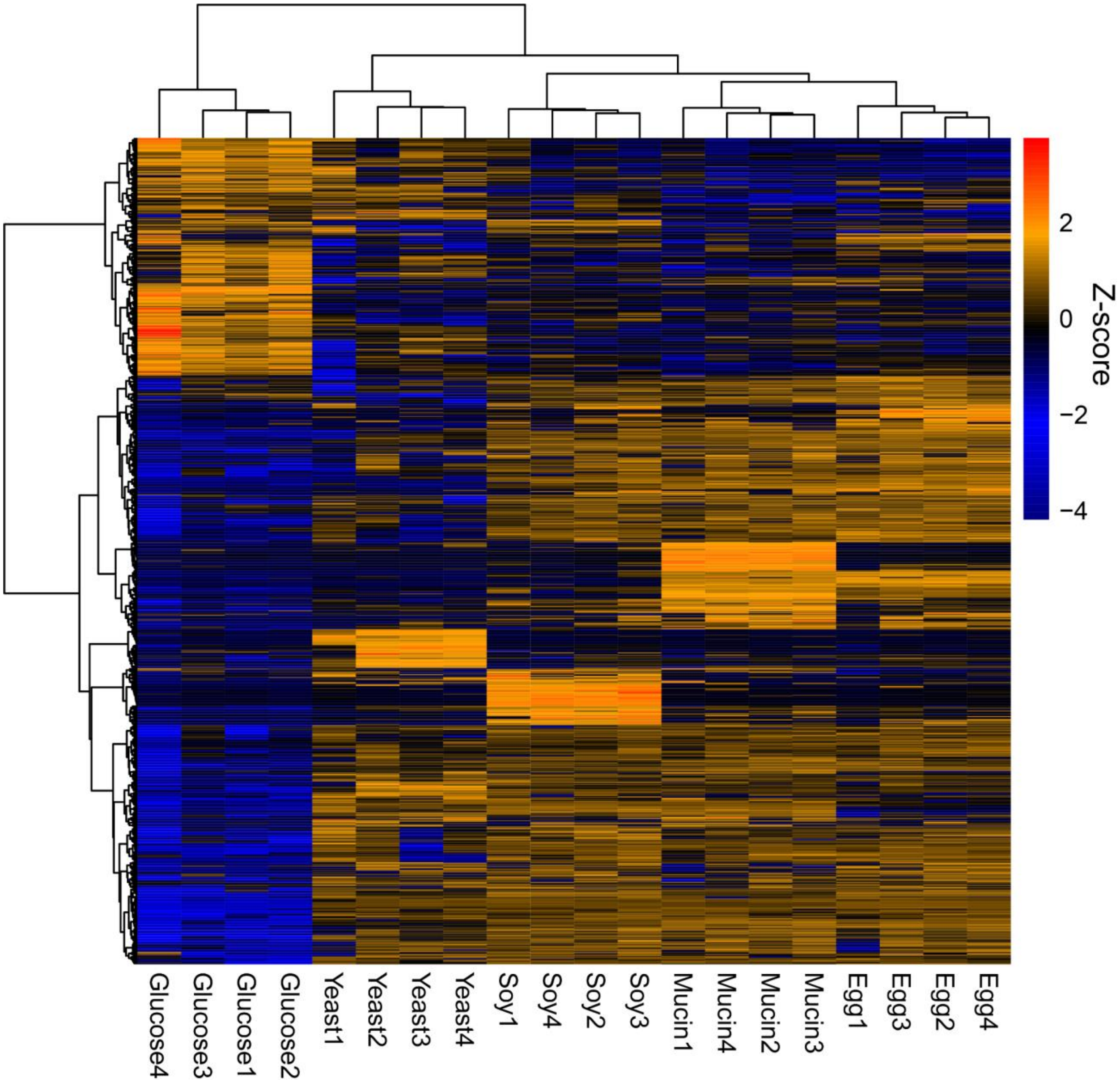

Supplementary Figure S20: Significantly different proteins in in vitro proteomes of *B. thetaiotaomicron* clustered by growth medium. Clustered heatmap of the in vitro proteomes of *B. thetaiotaomicron* after *Z* score standardization and removal of non-significant proteins after testing by ANOVA ($q<0.05$). We generated dendrograms using the ward clustering algorithm on euclidean distances.

References

1. Bosdriesz E, Molenaar D, Teusink B *et al.* How fast-growing bacteria robustly tune their ribosome concentration to approximate growth-rate maximization. *FEBS J* 2015;**282**:2029–44. 10.1111/febs.13258.

2. Fasnacht M, Polacek N. Oxidative stress in bacteria and the central dogma of molecular biology. *Front Mol Biosci* 2021;**8**. 10.3389/fmolb.2021.671037.

3. Tailford LE, Crost EH, Kavanaugh D *et al.* Mucin glycan foraging in the human gut microbiome. *Front Genet* 2015;**6**. 10.3389/fgene.2015.00081.

4. Hahnke RL, Meier-Kolthoff JP, García-López M *et al.* Genome-based taxonomic classification of bacteroidetes. *Front Microbiol* 2016;**7**.10.3389/fmicb.2016.02003

5. Cavaglieri CR, Nishiyama A, Fernandes LC *et al.* Differential effects of short-chain fatty acids on proliferation and production of pro- and anti-inflammatory cytokines by cultured lymphocytes. *Life Sci* 2003;**73**:1683–90. 10.1016/s0024-3205(03)00490-9.

6. Tedelind S, Westberg F, Kjerrulf M *et al.* Anti-inflammatory properties of the short-chain fatty acids acetate and propionate: a study with relevance to inflammatory bowel disease. *World J Gastroenterol* 2007;**13**:2826–32. 10.3748/wjg.v13.i20.2826.

7. Kleiner M, Thorson E, Sharp CE *et al.* Assessing species biomass contributions in microbial communities via metaproteomics. *Nat Commun* 2017;**8**:1558. 10.1038/s41467-017-01544-x.

8. Terrapon N, Lombard V, Drula É *et al.* PULDB: the expanded database of Polysaccharide Utilization Loci. *Nucleic Acids Res* 2018;**46**:D677–83. 10.1093/nar/gkx1022.
